# PHE1-based IgG-like antibody platform provides a novel strategy for enhanced T-cell immunotherapy

**DOI:** 10.3389/fimmu.2024.1415834

**Published:** 2024-06-11

**Authors:** Lingbin Wang, Haojie Jiang, Xuying Yin, Tingting Liang, Guoming Li, Chen Ding, Mina Yang, Lin Zhang, Junling Liu, Yanyan Xu

**Affiliations:** ^1^ Department of Biochemistry and Molecular Cell Biology, Key Laboratory of Cell Differentiation and Apoptosis of Chinese Ministry of Education, Shanghai Jiao Tong University School of Medicine, Shanghai, China; ^2^ Shanghai Synvida Biotechnology Co., Ltd, Shanghai, China

**Keywords:** bispecific antibody, T-cell engaging bsAb, T-cell immunotherapy, B cell mature antigen, PHE1-based bsAb

## Abstract

**Introduction:**

Bispecific antibodies (BsAbs) can simultaneously target two epitopes of different antigenic targets, bringing possibilities for diversity in antibody drug design and are promising tools for the treatment of cancers and other diseases. T-cell engaging bsAb is an important application of the bispecific antibody, which could promote T cell-mediated tumor cell killing by targeting tumor-associated antigen (TAA) and CD3 at the same time.

**Methods:**

This study comprised antibodies purification, Elisa assay for antigen binding, cytotoxicity assays, T cell activation by flow cytometry *in vitro* and xenogenic tumor model *in vivo*.

**Results:**

We present a novel bsAb platform named PHE-Ig technique to promote cognate heavy chain (HC)-light chain (LC) pairing by replacing the CH1/CL regions of different monoclonal antibodies (mAbs) with the natural A and B chains of PHE1 fragment of Integrin β2 based on the knob-in-hole (KIH) technology. We had also verified that PHE-Ig technology can be effectively used as a platform to synthesize different desired bsAbs for T-cell immunotherapy. Especially, BCMA×CD3 PHE-Ig bsAbs exhibited robust anti-multiple myeloma (MM) activity *in vitro* and *in vivo*.

**Discussion:**

Moreover, PHE1 domain was further shortened with D14G and R41S mutations, named PHE-S, and the PHE-S-based BCMA×CD3 bsAbs also showed anti BCMA^+^ tumor effect *in vitro* and *in vivo*, bringing more possibilities for the development and optimization of different bsAbs. To sum up, PHE1-based IgG-like antibody platform for bsAb construction provides a novel strategy for enhanced T-cell immunotherapy.

## Introduction

Bispecific antibodies (BsAbs) refer to the artificial antibodies that specifically bind to two antigens or epitopes at the same time (1). Since 1960, when the concept of bsAbs was first introduced, chemical coupling, two-hybrid tumor fusion, and genetic engineering techniques have been used to prepare bsAbs (2). In recent years, with the gradual maturation of recombinant antibody expression and purification technology *in vitro*, bsAbs have even shown a clear blowout trend. The simultaneous targeting of different specific antigenic epitopes has made bsAbs a hot topic in the field of pharmaceutical research and development, due to the fact that they make it possible to inhibit angiogenesis, decay tumor growth or even enhance tumor immunity with a single antibody at the same time (3–6). Besides, bsAbs could also serve as a bridge between two proteins or even two types of cells. For example, Emicizumab could bind to FX and FIX, promote the coagulation cascade and be used for the treatment of Hemophilia A (7). Blinatumomab is another marketed bsAbs, which binds to CD3 and CD19, bridges the T cell and the tumor cell of multiple myeloma and thus kills tumor cells (8). Other bispecific antibody (bsAb) application similar to Blinatumomab could be defined as bispecific T-cell engager (BiTE), which contains two different single-chain fragment variable (scFV) antibodies to two different targets and could promote T cell-mediated tumor cell killing by targeting tumor-associated antigen (TAA) and CD3 at the same time (9, 10).

BsAb consists of four different heavy chains and light chains, which gives rise to the high probability of the mis-assembly of light and heavy chains, making it difficult to purify the desired combination that we need (11). To solve this problem, some strategies have been developed. Among them, konb-in-holes (KIH) technology could reduce the probability of mismatch between two heavy chains of bsAbs by introducing the mutations in the CH3 domain on one heavy chain of bsAbs (12). CrossMab is a technique that interchanges the Fab region, VH-VL region or CH1-CL region of one side of bsAbs, which could increase the chance of correct matching between heavy chain and light chain (13). In addition, there are now bsAbs that do not contain the Fc region, and consist mainly of scFV forms of different antibodies or single-domain antibodies in tandem (14). Among which, BiTE expands the application of bsAbs that can be used in T-cell immunotherapy (15).

PHE1 is a natural structure of Integrin β2 subunit with A and B chains, consisting of the PSI, hybrid, and I-EGF1 domains (PDB: 2P28), which might replace CH1 and CL domain of antibodies and increase the probability of desired pairing of antibody heavy and light chains (16). Through analyzing multi-protein structures, it was found that the replacement of CH1 domain with PHE1 B chain and the replacement of CL domain with PHE1 A chain could form a stable heterodimer. This natural structure may avoid the mismatch of light and heavy chains in bsAbs and serve as an effective tool for designing a novel bispecific IgG-like antibody platform. At the same time, KIH technology is also used to avoid the problem of heavy chain mismatch. Here, we named it PHE-Ig technology. Currently, T-cell-engaging bsAb is an important application of bsAbs. Here, we initially verified the feasibility of PHE-Ig technology by synthesizing three different T-cell engaging bsAbs by PHE-Ig technology. One of these bsAbs, BCMA×CD3, was used as the primary target to verify that the BCMA×CD3 PHE1-based bsAb could mediate T cell killing of tumor cells both *in vivo* and *in vitro*.

In this study, we developed the PHE-Ig technology and found the PHE1-based bsAb synthesized by PHE-Ig technology can perform its function *in vivo* and *in vitro*. And we further shortened the structure of PHE1-A and B chains into PHE-S, which can also be used for bsAb production, bringing more possibilities for the development and optimization of different bsAbs. To sum up, PHE1-based IgG-like antibody platform for bsAb construction provides a novel strategy for enhanced T-cell immunotherapy.

## Methods

### Design of antibodies

We used the structure of IgG4 as the basic skeleton to construct bsAbs and used KIH technology to avoid heavy chain mis-assembly. Chain A and B of PHE1 have a natural heterodimer-like structure which has a disulfide bond (Cys^33^-Cys^447^). PHE1 A chain (23-122 amino acids) and PHE1 B chain (362-482 amino acids) of Integrin beta-2 were used to replace the CH1-CL domain to resolve light chain mismatch during the expression of bsAbs (PDB:2P28). The length of the A and B chains of PHE1 were further shortened and made mutations at D14G and R41S to obtain the PHE-S-Ig technology. The sequences of anti-CD19, anti-EGFR, anti-BCMA and anti-CD3 antibodies were derived from Blinatumomab, Panitumumab, Belantamab, Mosunetuzumab and OKT3.

### Purification of antibodies

The respective light and heavy chain plasmids were designed according to the antibody construction strategy. The corresponding plasmids were transiently transfected into CHO cells using electroporation. After electroporation, the cells were incubated in shake flasks at 37°C, 120 rpm, 8% CO_2_ for 24 h. And then, commercial supplements were added. Cells were cultured at 37°C for four days. The supernatant was collected by centrifugation and purified by protein A affinity chromatography. Afterwards, the purity of the proteins was examined by SDS-PAGE and SEC.

### Antigen binding

To assess the ability of antibodies binding to antigens, we used Enzyme-linked immunosorbent assay (ELISA). 96-well plates were coated with CD19, EGFR, BCMA and CD3 (Sino Biological Inc) overnight at 4°C and then blocked with 2% BSA for 1 hour at room temperature. BsAbs and control antibodies were diluted by 2% BSA and incubated for 1 hour at room temperature. Plates were washed three times with PBST (PBS with 0.005% Tween 20). Horseradish peroxidase (HRP)-conjugated anti-human IgG Fc antibodies (Thermo Fisher Scientific) were incubated for 1 hour at room temperature. TMB (Thermo Fisher Scientific) was used as substrates. Reaction was terminated by 2M H_2_SO_4_ and absorption was measured at a wavelength of 450 nM.

Flow cytometry was used to evaluate the ability of antibodies binding to antigens on cell surface. T cells or NCI-H929 were incubated with bsAbs and control antibodies for 1 hour at 4°C and then washed three times with FACS buffer. Alexa Fluor® 647 AffiniPure™ Alpaca Anti-human IgG (Jackson ImmunoResearch) was used to detect the bound antibodies. Flow cytometry was performed by using CytoFLEX LX (Beckman) and data were analyzed by using FlowJo (BD biosciences).

### Cytotoxicity assays and T-cell activation

Human peripheral blood mononuclear cells (PBMCs) were isolated from healthy donors by using Ficoll-Paque PLUS (Cytiva). And then, T cells were isolated by using EasySep™ Human T Cell Isolation Kit (Stemcell Technologies) from PBMCs. To evaluate the killing of NCI-H929, 1 × 10^5^ NCI-H929 cells/well were plated in 96-well plates (10:1 effector-to-target [E:T] ratio) with T cells and antibodies for 24 hours at 37°C. Killing of NCI-H929 was measure by quantifying lactate dehydrogenase (LDH) released from dead cells into the supernatant (LDH Assay Kit, Beyotime Biotechnology). TNF-α and IFN-γ were released and CD25 was expressed on T cell surface when T cells were activated. TNF-α and IFN-γ were measured using ELISA MAX™ Deluxe Set Human IFN-γ and ELISA MAX™ Deluxe Set Human TNF-α (BioLegend). CD25-postive T cells were analyze by flow cytometry.

### Xenogenic tumor model

NOD-scid-IL2Rgnull (NSG) mice were purchased from Shanghai Model Organisms Center, Inc. (Shanghai, China). NSG mice (8–10 weeks) were injected intravenously with 5 ×10^6^ NCI-H929- Luciferase cells (purchased from Shanghai Model Organisms Center, Inc.). Mice were injected intravenously with a mixture of 1 × 10^7^ human PBMC and antibodies (0.5 mg/kg) after five days. Antibodies (0.5 mg/kg) were administered every two days and mice were analyzed by using bioluminescence imaging (BLI) to track tumor burden. The Shanghai Jiao Tong University School of Medicine Animal Care and Use Committee approved the animal research.

## Results

### PHE-Ig technology can be effectively applied to synthesize PHE1-based bsAbs

**Figure 1:**
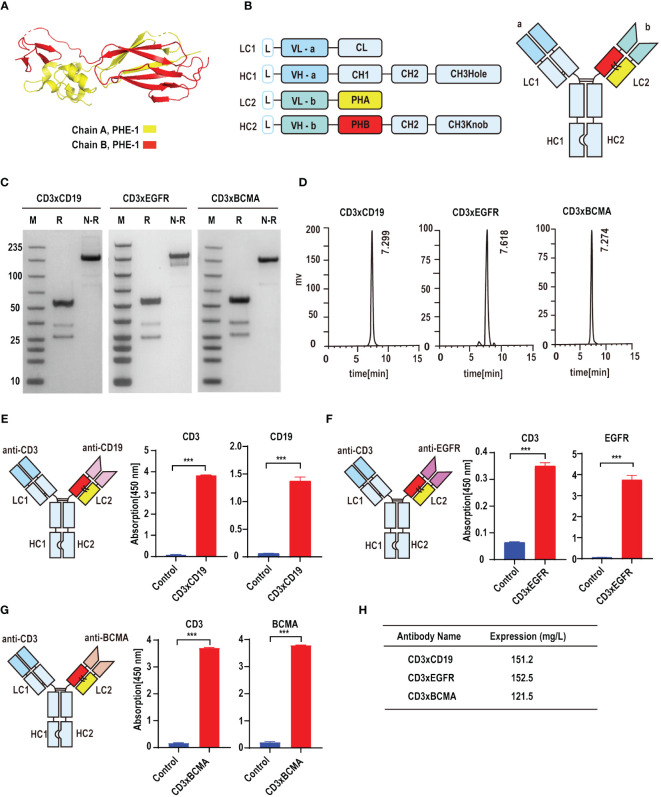
PHE-Ig technology can be effectively applied to synthesize PHE1-based bsAbs. **(A)** Protein structure of the A and B chains of PHE1, with the A chain represented in yellow and the B chain in red. **(B)** Schematic diagram of PHE1-based bsAbs includes a schematic of the light chain (LC) and heavy chain (HC) of PHE1-based bsAb and a schematic of the overall structure. PHA refers to the 23–122 amino acids of integrin beta-2 and PHB refers to a part of the 362–482 amino acids of integrin beta-2. **(C)** SDS-PAGE analysis of PHE1-based bsAb CD3×CD19, CD3×EGFR and CD3×BCMA under reducing or non-reducing conditions. M stands for marker, R for the reduced state, and N-R for the non-reduced case. **(D)** SEC analysis of PHE1-based bsAb CD3×CD19, CD3×EGFR and CD3×BCMA. **(E)** Schematic structure of CD3×CD19, and the ELISA testing binding of CD3×CD19 to CD3 and CD19 respectively. Mean ± SD, n = 3. ^***^P < 0.005. **(F)** Schematic structure of CD3×EGFR, and ELISA testing binding of CD3×EGFR to CD3 and EGFR respectively. Mean ± SD, n = 3. ^***^P < 0.005. **(G)** Schematic structure of CD3×BCMA, and ELISA testing binding of CD3×BCMA to CD3 and BCMA respectively. Mean ± SD, n = 3. ^***^P < 0.005. **(H)** The yields of CD3×CD19, CD3×EGFR and CD3×BCMA after purification.

On the basis of KIH technology, we used the A and B chains of PHE1 to replace the CH1-CL domains in order to ensure the effective production of bsAbs (**Figure 1A**). CD19, epidermal growth factor receptor (EGFR) and B cell maturation antigen (BCMA) are effective anti-tumor targets in clinical practice (17–20). To test whether PHE1-based bsAbs could be produced efficiently, we generated three T-cell-engaging PHE1-based bsAbs CD3×CD19, CD3×EGFR, and CD3×BCMA (**Figure 1B**). The anti-CD3 domain is selected for the variable domains on the unmodified side and the anti-tumor target domain (anti-CD19, anti-EGFR, anti-BCMA) is assembled in the variable domains on the modified (PHE1) side. The corresponding plasmids were designed to be co-expressed in CHO cells and then the corresponding bsAbs were obtained respectively. The three bsAbs are purified by protein A affinity chromatography with yields of 151.2 mg/L (CD3×CD19), 152.5 mg/L (CD3×EGFR), and 121.5 mg/L (CD3×BCMA) (**Figure 1H**). The predicted molecular mass of CD3×CD19 and CD3×BCMA was 149 kDa, and that of CD3×EGFR was 148 kDa. SDS-PAGE analysis showed a band at approximately 150 kDa under non-reducing conditions, representing the PHE1-based bsAbs successfully assembled (**Figure 1C**). SEC Analysis of CD3×CD19 and CD3×BCMA showed a single peak, and that of CD3×EGFR showed a single peak with low levels of aggregates (**Figure 1D**). CD3×CD19 and CD3×BCMA were predominantly (>95%) monomeric in content, CD3×EGFR was >85% monomeric in content. The above results indicated that PHE1-based bsAbs can be efficiently produced. Finally, CD3, CD19, EGFR and BCMA were used as fixed antigens respectively, and ELISA binding results showed that CD3×CD19, CD3×EGFR, and CD3×BCMA could specifically bind to their respective targets (**Figures 1E–G**). The above results indicated that the PHE-Ig technology can be applied to synthesize different bsAbs, suggesting the general applicability of PHE-Ig technology.

### Functional characterization of BCMA×CD3 PHE1-based bsAb

**Figure 2:**
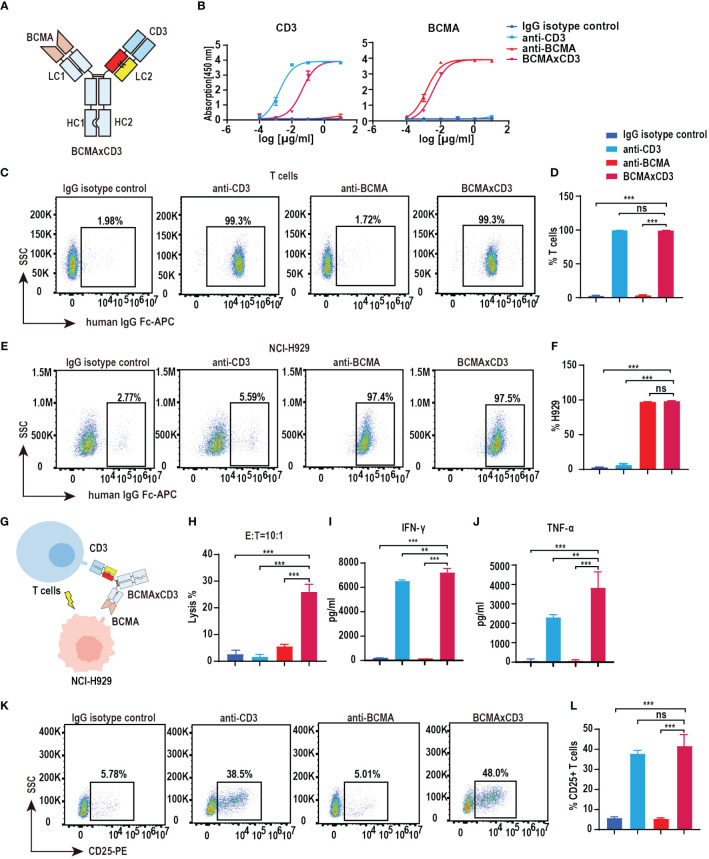
Functional characterization of BCMA×CD3 PHE1-based bsAb. **(A)** Schematic structure of BCMA×CD3. **(B)** ELISA testing binding of BCMA×CD3 specifically to CD3 and BCMA, respectively. Parental mAbs were included as control. Mean ± SD, n = 3. **(C, D)** Flow cytometry analysis binding of BCMA×CD3 to human T (CD3^+^) cells. Parental mAbs were included as control. Mean ± SD, n = 3. Ns, P > 0.05; ^***^P < 0.005. **(E, F)** Flow cytometry analysis binding of BCMA×CD3 to NCI-H929 (BCMA^+^) cells. Parental mAbs were included as control. Mean ± SD, n = 3. Ns, P > 0.05; ^***^P < 0.005. **(G)** The pattern diagram of BCMA×CD3 mediated T cells killing NCI-H929 cells. **(H)** NCI-H929 cells were co-incubated with T cells (E:T=10:1) and BCMA×CD3 (10µg/mL) for 24 hours. The levels of tumor killing was indicated by measuring the release of lactated hydrogenase (LDH). Mean ± SD, n = 3. ^***^P < 0.005. **(I, J)** Cytokines (IFN-γ and TNF-α) released from T cells cocultured with the NCI-H929 cells in the presence of BCMA×CD3 (10µg/mL) for 24 hours. Mean ± SD, n = 3. ^**^P < 0.01; ^***^P < 0.005. **(K, L)** H929 cells, T cells (E:T=1:10) and BCMA×CD3 (10 μg/mL) were co-incubated for 24 hours and then the level of CD25^+^ T cells was detected by flow cytometry. Parental mAbs were included as control. Mean ± SD, n = 3. Ns, P > 0.05; ^***^P < 0.005.

We have verified the feasibility of the PHE-Ig technique for synthesizing bsAbs. BCMA is highly selectively expressed in malignant plasma cells (PCs) and is an effective therapeutic target for MM (14, 21). Therefore, BCMA×CD3 (anti-CD3 variable domain on the PHE1-side of the antibody) was selected to further assess the function characterization of PHE1-based bsAbs (**Figure 2A**). ELISA results showed that BCMA×CD3 specifically bound to BCMA similar to anti-BCMA mAb and bound to CD3 as anti-CD3 mAb, both in a concentration-dependent manner (**Figure 2B**). BCMA×CD3 could also bind to human T (CD3^+^) and NCI-H929 cells (BCMA^+^, human myeloma cell) similar to corresponding parental mAbs, which were analyzed by flow cytometry (**Figures 2C–F**). Moreover, BCMA×CD3 was able to mediate T cell cytotoxicity against NCI-H929 (**Figures 2G, H**), which was accompanied with the production of TNF-α, IFN-γ and IL2 cytokines (**Figures 2I, J**; **Supplementary Figure 2E**), the expression of the activation and proliferation marker on T cells (**Figures 2K, L**; **Supplementary Figures 2A–D**). In conclusion, we used PHE-Ig technology to generate BCMA×CD3 PHE1-based bsAbs, which can effectively induce T-cell-mediated tumor cell killing and activation of T cells. These results demonstrated that PHE-Ig technology can be used for the production of T-cell-engaging bsAb.

### PHE-S-Ig technology is able to form functional BCMA×CD3 bsAb

**Figure 3:**
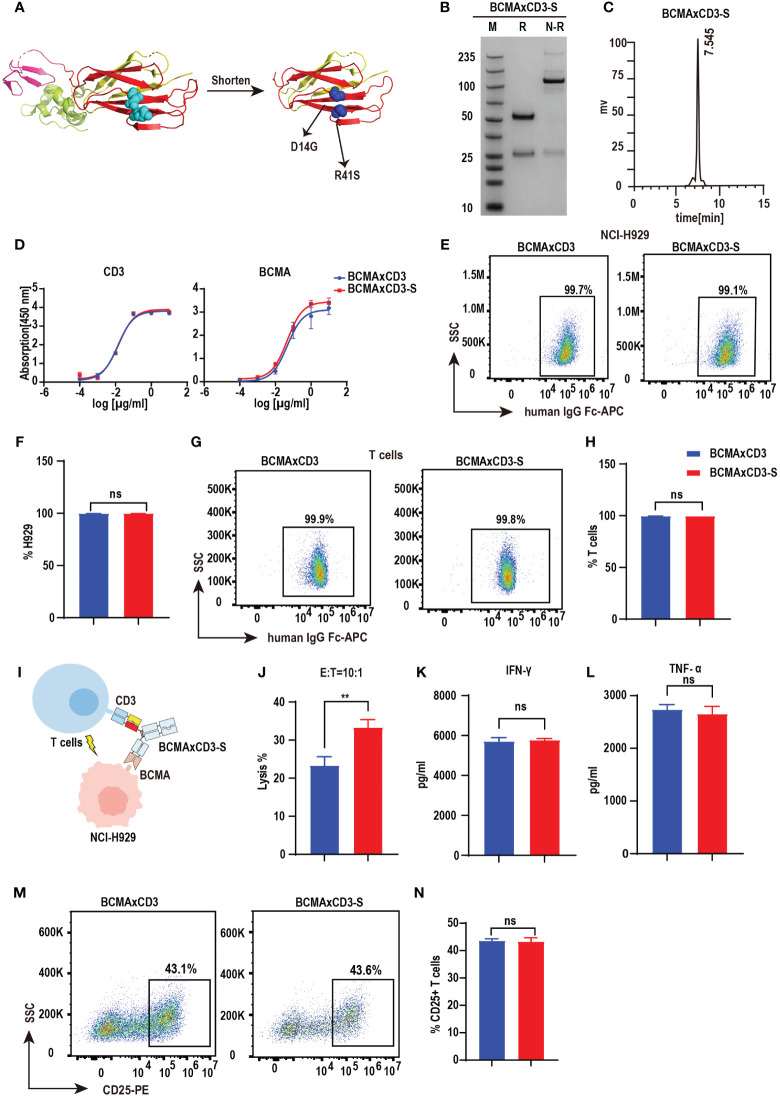
PHE-S-Ig technology is able to form functional BCMA×CD3 bsAb. **(A)** The structures of the shortened and mutated A and B chains of PHE1 protein, named PHE-S, the shortened A chain represented in yellow, the shortened B chain in red, and the mutated amino acids in blue. **(B)** SDS-PAGE analysis of BCMA×CD3-S. **(C)** SEC analysis of BCMA×CD3-S. **(D)** ELISA testing binding of BCMA×CD3-S to CD3 and BCMA proteins, respectively. Parental mAbs were included as control. Mean ± SD, n = 3. **(E, F)** Flow cytometry analysis binding of BCMA×CD3-S to NCI-H929 (BCMA^+^) cells. BCMA×CD3 were included as control. Mean ± SD, n=3. Ns, P > 0.05. **(G, H)** Flow cytometry analysis binding of BCMA×CD3-S to human T (CD3^+^) cells. BCMA×CD3 were included as control. Mean ± SD, n=3. Ns, P > 0.05. **(I)** The pattern diagram of BCMA×CD3-S mediated T cells killing NCI-H929 cells. **(J)** NCI-H929 cells were co-incubated with T cells (E:T=10:1) and BCMA×CD3-S (10μg/mL) for 24 hours. The levels of tumor killing was indicated by measuring the release of lactated hydrogenase (LDH). Mean ± SD, n = 3. ^**^P < 0.01. **(K, L)** Cytokines (IFN-γ and TNF-α) released from T cells cocultured with the NCI-H929 cells in the presence of BCMA×CD3-S (10µg/mL) for 24 hours. Mean ± SD, n = 3. Ns, P > 0.05. **(M, N)** H929 cells, T cells (E:T=1:10) and BCMA×CD3-S (10 µg/mL) were co-incubated for 24 hours and then the level of CD25^+^ T cells was detected by flow cytometry. Mean ± SD, n = 3. Ns, P > 0.05.

Bispecific antibodies contain two distinct, variable regions that recognize different antigenic epitopes. Although the substitution of the PHE-1 for one of the CH1-CL pairs in the antibody can be used to form stable and functional bispecific antibodies, other possibilities based on the PHE-1 structure are still needed for the development and optimization of bispecific antibodies. We thus designed a series of engineering schemes to shorten the protein structure of PHE1. In the new PHE-S-Ig technology, we shortened the length of PHE1, made mutations at D14G, R41S and obtained a new stable heterodimer, which was further used to replace the CH1-CL regions of antibodies with KIH technology, producing a novel PHE-S-based bsAb structure (**Figure 3A**). To test its efficacy, we synthesized BCMA×CD3-S using the same variable domains as BCMA×CD3 based on PHE-S-Ig technology. Plasmids of the respective corresponding light and heavy chains were designed and co-transfected into CHO cells, and BCMA×CD3-S were obtained by protein A affinity chromatography purification. The yield of BCMA×CD3-S was 231.1 mg/L, comparable to PHE1-based BCMA×CD3 bsAb, but higher to crossmab-based BCMA×CD3 bsAb (**Supplementary Figures 1A–C**). SDS-PAGE analysis of BCMA×CD3-S showed a band at 100–150 kDa under non-reducing conditions, consistent with its predicted molecular mass (**Figure 3B**). SEC result exhibited a single peak of BCMA×CD3-S (**Figure 3C**). BCMA×CD3-S bsAb bound to BCMA and CD3 in a concentration-dependent manner (**Figure 3D**) and could specifically combined to T cells and NCI-H929 (**Figures 3E–H**) as BCMA×CD3. The killing effect of BCMA×CD3-S mediated T cells on NCI-H929 cells was also determined (10 µg/mL, E:T=10:1). BCMA×CD3-S has an increased tumor cell killing rate, compared to BCMA×CD3 *in vitro* (**Figures 3I, J**). In the process of BCMA×CD3-S-mediated T cell killing of NCI-H929, the cytokines TNF-α, IFN-γ and IL2 were also produced (**Figures 3K, L**; **Supplementary Figure 3C**) and the activation and proliferation marker was expressed on T cells (**Figures 3M, N**; **Supplementary Figures 3A, B, D, E**). Moreover, the fluorescence micrography results showed that both BCMA × CD3 and BCMA × CD3-S bsAbs could bring T cells closer to H929 cells compared with control group (**Supplementary Figure 3F**). Sum up, BCMA×CD3-S synthesized by PHE-S-Ig technique were also effective to induce T cell-mediated anti-tumor than BCMA×CD3 *in vitro.*

### The BCMA×CD3 and BCMA×CD3-S showed anti-MM efficacy in NCI-H929-mediated MM model

**Figure 4:**
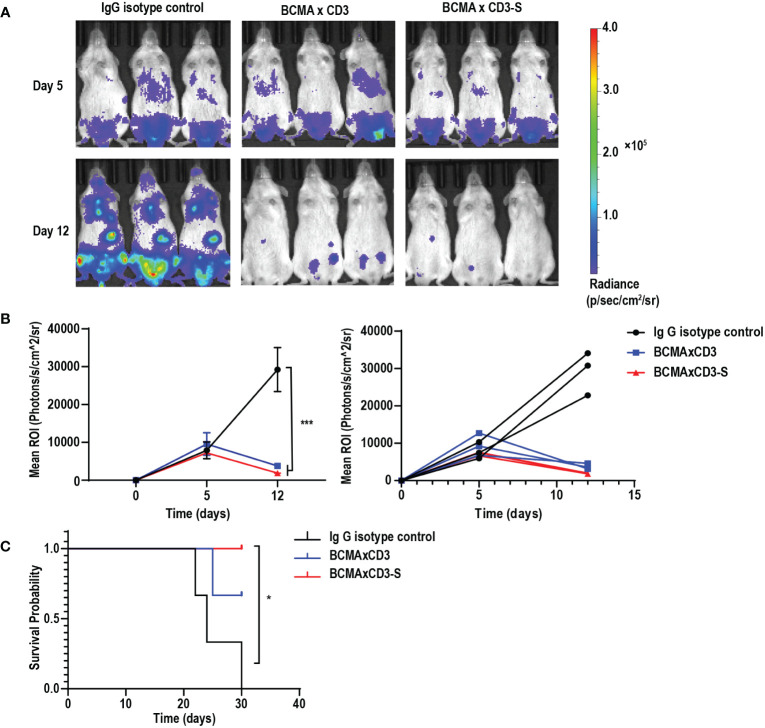
BCMA×CD3 and BCMA×CD3-S showed significant antitumor efficacy in NCI-H929-mediated MM model. **(A)** NSG mice that had been injected intravenously with NCI-H929-Luc cells for five days were engrafted intravenously with PBMC and received intravenously BCMA×CD3 and BCMA×CD3-S every 2 days. The bioluminescence imaging (BLI) showed their antitumor effects. **(B)** Quantification of NCI-H929 burden indicated by the radiance detected in the region of interest with group values. ROI, the region of interest. Mean ± SD, n = 3. ^***^P < 0.005. **(C)** Kaplan Myer survival curve of mice. Significance measured by Log-rank Mantel-Cox test. n = 3, *P < 0.05.

To assess the anti-tumor efficacy of BCMA×CD3 and BCMA×CD3-S *in vivo*, we established the NCI-H929-mediated MM models. NCI-H929-luciferase-positive cells were injected intravenously into NSG mice. These mice received a mixture of antibodies and PBMC after five days, followed by injections of antibodies every two days. Both BCMA×CD3 and BCMA×CD3-S significantly suppressed tumor growth (**Figures 4A, B**), as well as had a protective effect on the survival of NCI-H929-bearing NSG mice (**Figure 4C**). The result indicated that the PHE-Ig and PHE-S-Ig technology may enable T-cell-engaging bsAb to perform corresponding functions *in vivo*, as manifested in this study by *in vivo* mediating T cell killing of tumor cells. To sum up, both PHE-Ig and PHE-S-Ig technology enable bsAbs to exert their biological functions *in vivo*.

## Discussion

In 1960, Nisonoff et al. introduced the concept of bispecific antibodies (bsAbs) for the first time, opening up a new direction in antibody research and development (22, 23). The treatment of tumors was the first promising applications of bsAbs. Compared with monoclonal antibodies, bsAbs with two different antigen-binding sites hold great therapeutic promise (24, 25). Catumaxomab and Blinatumomab were the first two bsAbs that was approved for the treatment of malignant ascites and B-cell acute lymphoblastic leukemia (26, 27). Both of which include an anti-CD3 side, and another anti-TAA side, allowing for the T-cell mediated anti-tumor immunotherapy. In addition, bsAbs can also target different antigens on the surface of the same cell at the same time, thus enhancing the anti-tumor specificity and effectiveness of the antibody drug (28). For instance, Amivantamab (EGFR×MET) designed for non-small-cell lung cancer exhibit impressive clinical results (29). As bsAbs continue to be investigated, their value for other applications such as the treatment of non-tumor diseases is also continuously developed (30). Emicizumab was designed for the treatment of hemophilia A which can to bind specifically to FIXa and FX factors, mimicking the activity of FVIIIa (31). Currently, the application value of bsAbs is constantly being explored and it is of great significance to develop more effective bsAbs platforms (32).

BsAbs platforms have been developed and applied, which are mainly categorized into two types: bsAb platforms without Fc fragments and bsAb platforms with Fc fragments (28). Fc fragment-free bsAb platforms such as Bispecific T cell engager (BiTE) and DART have the advantage of avoiding the mismatch between light and heavy chains and increasing bsAbs permeability due to their small molecular mass, but they have short half-life and cannot be used as a universal technology (29, 33). Fc fragment-containing technology platforms are capable of decreasing the light and heavy chain mismatch, along with a longer half-life and basic effects such as antibody-dependent cell-mediated cytotoxicity (ADCC) and complement dependent cytotoxicity (CDC) (34). They can be further divided into structural symmetry and asymmetric IgG-like bsAbs. Most asymmetric bsAbs are from two different mAbs, normally containing two heterologous heavy chains and two heterologous light chains. However, the difficulty in the production of bsAbs is that the randomly paired heterologous heavy chains and light chains can lead to 90% misassembled species, such as homodimers and molecules with mis-paired light chains (1). Various strategies have been established to enforce desired chain pairing, such as KIH, WuXiBody and CrossMab technologies (12, 13, 35).

PHE1-based bsAb is a novel and efficient bsAb platform, which have a higher production yield than crossmab technology in this study. We replaced the CH1-CL structural domain of human IgG4 with the A and B chains of PHE1 based on the KIH technology to make mAb sequence pair assembled into the bispecific construct. It enables mAb sequence pair to be assembled into a desired bispecific pair using the PHE-Ig technique and had relatively high yields of bsAbs (36, 37). By analysis of the SDS-PAGE and SEC, it was found that PHE1-based bsAb synthesis had low levels of mis-pairs and aggregation. Therefore, introduction of PHE1 fragment of Integrin β2 constant domain into bsAbs construction not only promotes desired chain pairing but also enhances the production and purities of bsAbs. Notably, when assembling the bsAbs using our PHE-Ig technique, the different assembly strategies using PHE-Ig technology may result in different yield rates of bsAbs.

Currently, T-cell engaging bsAbs have been continuously studied intensively, and some other encouraging achievements have been made, such as molecules like Blinatumomab, an anti-CD19/CD3 bsAb, and Teclistamab, an anti-BCMA/CD3 bsAb (38–40). Our PHE1-based anti-BCMA×CD3 bsAbs function as mediators of T cell killing of tumor cells both *in vivo* and *in vitro*, demonstrating the effectiveness of employing PHE1-based bsAb at both the cellular and animal levels. As mentioned above, we also shortened the PHE-Ig structure to obtain the PHE-S-Ig structure, which brings a possibility for further development and optimization of bispecific antibodies. In this study, BCMA×CD3-S bsAb treated T cells have a stronger lysis effect than BCMA×CD3 bsAb *in vitro*. Since the sequence of the variable region of the antibodies and the construction strategy of BCMA×CD3-S and the parental antibody BCMA×CD3 were not changed, the difference in lysis effect of the two may be due to the shortened structure of the PHE-S being more beneficial compared to the natural structure of the PHE1 replacing the CH1-CL structure for this BCMA×CD3 bsAb. However, whether PHE-S-Ig technology could be more efficient than PHE-Ig technology in other bsAbs model, such as CD19×CD3 or EGFR×CD3, requires more studies.

In conclusion, we established that the PHE-Ig technique can be used as a general technique for synthesizing bsAbs, and based on this, we shortened it to obtain the PHE-S-Ig technique. PHE-Ig technology can be used as a platform to synthesize bsAbs in a generalized manner, capable of synthesizing different bsAbs based on different specific variable domains. And the bsAbs synthesized using PHE-Ig and PHE-S-Ig technologies have biological activities *in vitro* and *in vivo*. Further experiments need to be taken to prove the safety of its therapeutic application.

## Data availability statement

The original contributions presented in the study are included in the article/**Supplementary Material**. Further inquiries can be directed to the corresponding authors.

## Ethics statement

Ethical approval was not required for the studies on humans in accordance with the local legislation and institutional requirements because only commercially available established cell lines were used. The animal study was approved by The Shanghai Jiao Tong University School of Medicine Animal Care and Use Committee. The study was conducted in accordance with the local legislation and institutional requirements.

## Author contributions

LW: Writing – review & editing, Writing – original draft, Validation, Supervision, Software, Project administration, Methodology, Investigation, Formal analysis, Data curation, Conceptualization. HJ: Writing – review & editing, Writing – original draft, Validation, Supervision, Software, Project administration, Methodology, Investigation, Formal analysis, Data curation, Conceptualization. XY: Writing – original draft, Software, Investigation, Conceptualization. TL: Writing – original draft, Supervision, Methodology, Data curation. GL: Writing – original draft, Supervision, Methodology, Data curation. CD: Writing – original draft, Supervision, Methodology, Data curation. MY: Writing – original draft, Formal analysis, Data curation, Conceptualization. LZ: Writing – original draft, Formal analysis, Data curation, Conceptualization. JL: Writing – review & editing, Writing – original draft, Visualization, Supervision, Software, Resources, Methodology, Investigation, Funding acquisition, Data curation, Conceptualization. YX: Writing – review & editing, Writing – original draft, Visualization, Validation, Supervision, Software, Resources, Project administration, Methodology, Investigation, Funding acquisition, Formal analysis, Data curation, Conceptualization.
