# Supplementary material for: PHE1-based IgG-like antibody platform provides a novel strategy for enhanced T-cell immunotherapy
Source: Front Immunol. 2024 Jun 11;15:1415834. doi: 10.3389/fimmu.2024.1415834 (PMC11201533; doi:10.3389/fimmu.2024.1415834)
Supplement: Supplementary file 1 [file DataSheet_1.pdf]

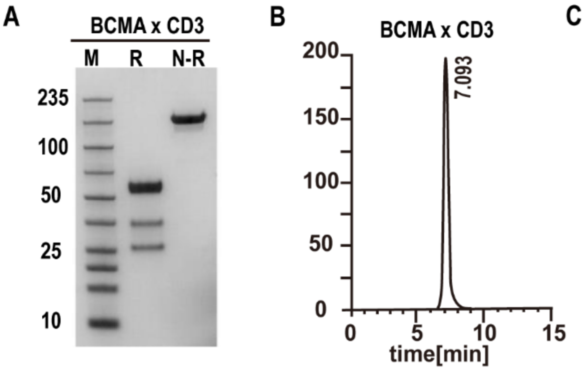

C

Antibody Name	Expression(mg/L)
BCMAxCD3	210.5
BCMAxCD3-S	231.1
BCMAxCD3-crossmab	155.6

Supplemental Figure 1. Analysis of BCMA×CD3 purification results.

(A) SDS-PAGE analysis of BCMA×CD3.

(B) SEC analysis of BCMA×CD3.

(C) The yields of BCMA×CD3, BCMA×CD3-S and BCMA×CD3-crossmab after purification.

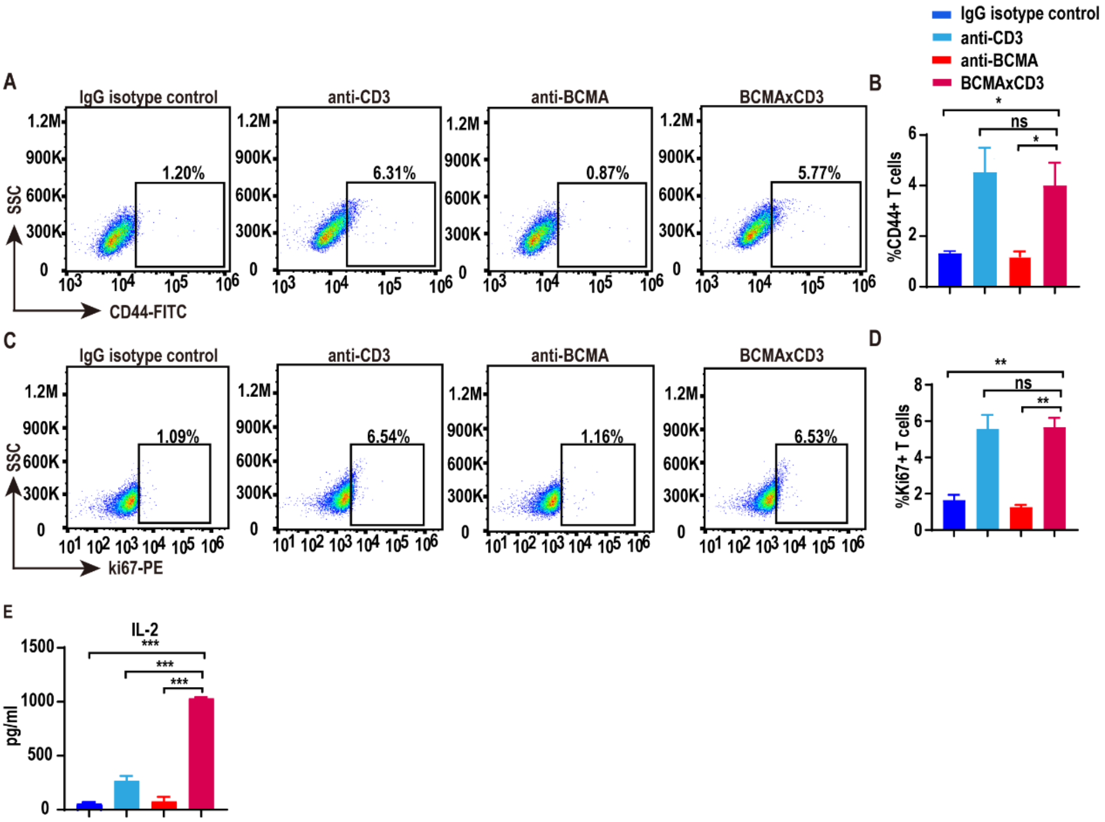

Supplemental Figure 2. BCMA×CD3 enhances T cell responses.

(A-B) H929 cells, T cells (E:T=1:5) and BCMA×CD3 (10 μg/mL) were co-incubated for 36 hours and then the level of $CD44^+$ T cells was detected by flow cytometry. Mean

± SD, n = 3. Ns, $P > 0.05$, *$P < 0.05$.

(C-D) H929 cells, T cells (E:T=1:5) and BCMA×CD3 (10 μg/mL) were co-incubated for 36 hours and then the level of $ki67^{+}$ T cells was detected by flow cytometry. Mean ± SD, n = 3. Ns, $P > 0.05$, **$P < 0.01$.

(E) Cytokines (IL-2) released from T cells cocultured with the NCI-H929 cells in the presence of BCMA×CD3 (10μg/mL) for 24 hours. Mean ± SD, n = 3. ***$P < 0.005$.

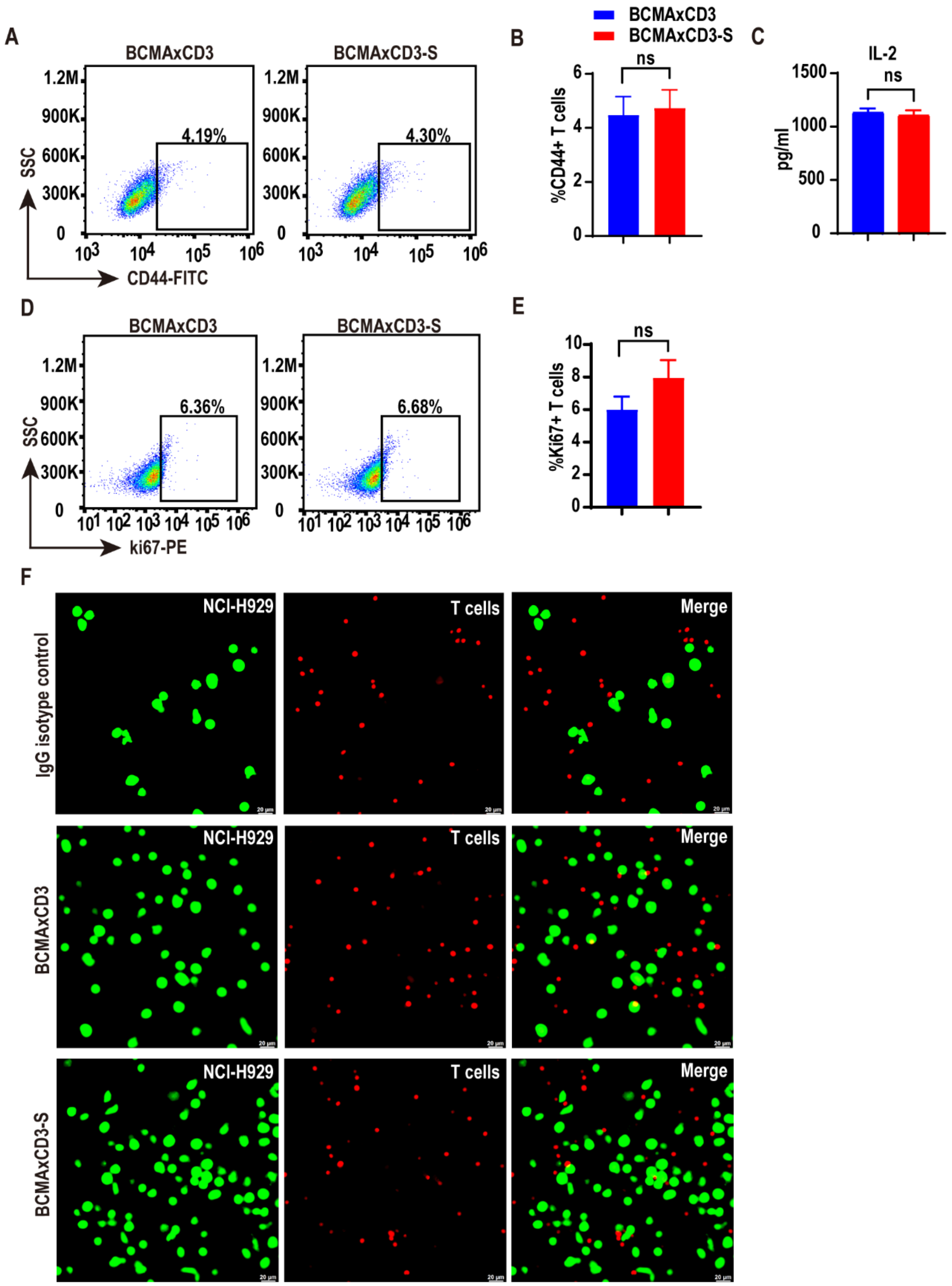

Supplemental Figure 3. BCMA×CD3-S enhances T cell responses as well as BCMA×CD3.

(A-B) H929 cells, T cells (E:T=1:5) and BCMA×CD3-S (10 μg/mL) were co-incubated for 36 hours and then the level of $CD44^+$ T cells was detected by flow cytometry. Mean ± SD, n = 3. Ns, $P > 0.05$.

(C) Cytokines (IL-2) released from T cells cocultured with the NCI-H929 cells in the

presence of BCMA×CD3-S (10μg/mL) for 24 hours. Mean ± SD, n = 3. Ns, $P > 0.05$.

(D-E) H929 cells, T cells (E:T=1:5) and BCMA×CD3-S (10 μg/mL) were co-incubated for 36 hours and then the level of $ki67^{+}$ T cells was detected by flow cytometry. Mean ± SD, n = 3. Ns, $P > 0.05$.

(F) H929 cells, T cells (E:T=1:2) and bsAb (10 μg/mL) were co-incubated for 9 hours. The location of NCI-H929 and T cells was observed under the microscope.
